# β-Ga_2_O_3_ Thin Films via an Inorganic Sol–Gel Spin Coating: Preparation and Characterization

**DOI:** 10.3390/nano15040277

**Published:** 2025-02-12

**Authors:** Hai Zhang, Dingyuan Niu, Junbiao Yang, Xiaoyang Zhang, Jun Zhu, Wencai Li

**Affiliations:** 1 School of Science, Inner Mongolia University of Technology, Hohhot 010051, China; 2 School of Physical Science and Technology, Inner Mongolia University, Hohhot 010021, China

**Keywords:** inorganic solution reaction, β-Ga_2_O_3_, spin coating, bandgap, transmittance

## Abstract

β-Ga_2_O_3_ holds significant promise for use in ultraviolet (UV) detectors and high-power devices due to its ultra-wide bandgap. However, the cost-effective preparation of large-area thin films remains challenging. In this study, β-Ga_2_O_3_ thin films are prepared using an inorganic solution reaction spin-coating method followed by post-annealing. The structures, surface morphologies, and optical properties of the films are then characterized using X-ray diffraction, scanning electron microscopy, and ultraviolet–visible spectrophotometry. A low-cost Ga metal was used to produce NH_4_Ga(SO_4_)_2_, which was then converted into a precursor solution and spin-coated onto sapphire and quartz substrates. Ten cycles of spin coating produced smoother films, although higher annealing temperatures induced more cracks. The films on the (0001) sapphire subjected to spin-coating and preheating processes that were repeated for ten cycles, followed by annealing at 800 °C, had a preferred orientation in the [–201] direction. All the films showed high transmittances of 85% in ultraviolet–visible light with wavelengths above 400 nm. The films on the (0001) sapphire substrate that were annealed at 800 °C and 1000 °C exhibited bandgaps of 4.8 and 4.98 eV, respectively. The sapphire substrates demonstrated a superior compatibility for high-quality Ga_2_O_3_ film fabrication compared to quartz. This method offers a cost-effective and efficient approach for producing high-quality β-Ga_2_O_3_ films on high-temperature-resistant substrates with promising potential for optoelectronic applications.

## 1. Introduction

β-Ga_2_O_3_ is considered a promising candidate for solar-blind photodetectors and high-power devices due to its desirable properties, including a large bandgap (4.9 eV) and excellent chemical, mechanical, and thermal stability [1,2]. Various forms of β-Ga_2_O_3_, including bulk [3,4,5,6], thin films [7,8,9,10,11,12], and nanostructures [13,14,15,16,17,18,19], have been synthesized and studied.

Many methods have been used for the deposition of Ga_2_O_3_ thin films, such as metal–organic chemical vapor deposition (MOCVD) [7,20,21,22,23,24], halide vapor-phase epitaxy (HVPE) [25,26,27], molecular beam epitaxy (MBE) [11,28,29], low-pressure chemical vapor deposition (LPCVD) [10,12,30,31,32,33], pulsed laser deposition (PLD) [34,35], magnetron sputtering [36,37], and plasma-enhanced chemical vapor deposition (PECVD) [38,39]. However, these methods often require specific reactions, high-vacuum conditions, or expensive source materials. In terms of practical applications, thin films are considered to have greater benefits than thicker films due to their compatibility for device manufacturing.

The solution sol–gel method has been widely used to prepare gallium oxide materials due to its simple equipment requirements, low cost, and high efficiency, as well as the ease of obtaining large-area homogeneous films when using this method [8,9,17,40,41,42]. However, the Ga_2_O_3_ films discussed in previous reports were regarded as either polycrystalline or of poor quality. Zhu et al. conducted a systematic study on the effects of various preheating temperatures on the crystal qualities and surface morphologies of β-Ga_2_O_3_ films using sol–gel spin coating followed by post-annealing, and the obtained epitaxial films were found to be oriented in the (–201) direction [40,42]. While gallium nitrate hydrate [Ga(NO_3_)_3_·xH_2_O] has often been used as a precursor, including in the aforementioned studies, the price of gallium salts remains relatively expensive.

In this study, we prepared β-Ga_2_O_3_ thin films deposited on (0001) sapphire substrates using an inorganic solution reaction spin-coating method followed by post-annealing. Low-cost Ga metal was used to produce NH_4_Ga(SO_4_)_2_, which was subsequently converted into a precursor solution. The effects of different annealing temperatures and numbers of spin-coating repetitions on the crystal qualities and surface morphologies of the β-Ga_2_O_3_ thin films were then systematically studied. We further assessed the effects of sapphire and quartz substrates on the transmittance and bandgaps of β-Ga_2_O_3_ thin films.

## 2. Materials and Methods

**Figure 1:**
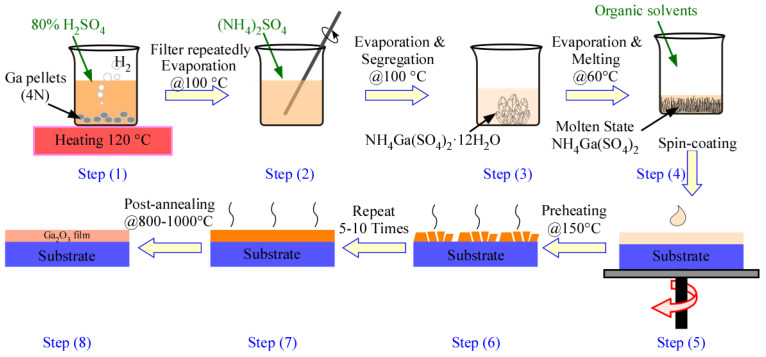
Schematic illustration of the Ga_2_O_3_ films prepared using the solution spin-coating method with a post-annealing process. Step (1): reaction between Ga and H_2_SO_4_. Step (2): repeated evaporation and filtration. Step (3): repeated evaporation and segregation to form NH_4_Ga(SO_4_)_2_·12H_2_O crystals. Step (4): evaporation, melting, and addition of an organic solvent to form the precursor solution. Step (5): spin coating of the solution on a substrate. Step (6): preheating at 150 °C. Step (7): spin-coating and preheating process consisting of 5–10 cycles. Step (8): post-annealing at 800–1000 °C to produce the final Ga_2_O_3_ film.

**Figure 2:**
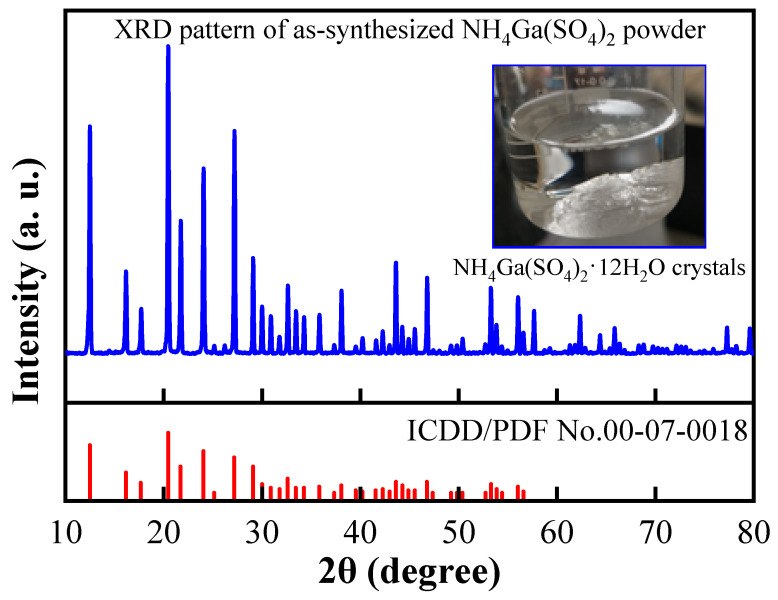
X-ray diffraction (XRD) patterns of the as-synthesized NH_4_Ga(SO_4_)_2_ powder, where the inset image shows the as-synthesized NH_4_Ga(SO_4_)_2_·12H_2_O crystals. Note: ICDD/PDF stands for International Centre of Diffraction Data/Powder Diffraction File.

The deposition process of the Ga_2_O_3_ films obtained using the solution spin-coating method presented in this work is shown in Figure 1. First, Ga metal pellets (with a purity of 99.99% and size of 3–5 mm, Zhongnuo Advanced Material (Beijing) Technology Co., Ltd., Beijing, China) and H_2_SO_4_ (80%, analytical grade, Tianjin Shengao Chemical Reagent Co., Ltd., Tianjin, China) were combined in a beaker and heated to 120 °C. An excessive amount of H_2_SO_4_ was maintained to ensure that the Ga metal fully reacted and dissolved. During heating, small hydrogen bubbles gradually formed on the Ga surface, while white precipitates of Ga_2_(SO_4_)_3_ started to form in the solution. A few yellow precipitates also appeared, likely due to sulfur impurities from the uneven temperature or excess H_2_SO_4_, and these impurities were removed through repeated filtration (Step 1 in Figure 1). The white Ga_2_(SO_4_)_3_ solid obtained from filtration was then dissolved in deionized water. Once fully dissolved, analytical grade (NH_4_)_2_SO_4_ (Tianjin Kemiou Chemical Reagent Co., Ltd., Tianjin, China) was added, after which more deionized water was added, and then the mixture was evaporated, filtered, and washed multiple times with deionized water (Step 2 in Figure 1). Following evaporation and cooling to room temperature, transparent NH_4_Ga(SO_4_)_2_·12H_2_O crystals (inset of Figure 2) formed (Step 3 in Figure 1). These crystals were then continuously heated at a temperature above 60 °C to evaporate the water, which caused the crystals to melt into a viscous molten liquid. Continued heating eventually turned this liquid into a white powder composed of NH_4_Ga(SO_4_)_2_. Ethylene glycol monomethyl ether (EGME and CH_3_OCH_2_CH_2_OH, AR, Shanghai Macklin Biochemical Technology Co., Ltd., Shanghai, China) and a small amount of polyacrylamide(AR, Shanghai Macklin Biochemical Technology Co., Ltd., Shanghai, China) were added to the powder as a solvent and a thickener, respectively, to improve its adhesion to the substrates in the subsequent film formation process. After thorough stirring, the mixture was left to precipitate into a gel (Step 4 in Figure 1). Quartz and c-sapphire substrates (10 × 10 mm) were subsequently prepared and ultrasonically cleaned with acetone, isopropanol, and deionized water for 10 min each. Then, spin coating was executed in three stages: stage 1 at 400 rpm for 10 s, stage 2 at 2500 rpm for 20 s, and stage 3 at 6000 rpm for 30 s (Step 5 in Figure 1). After each coating process, the substrate was preheated at 150 °C for 10 min (Step 6 in Figure 1). This process was then repeated for five and ten cycles to ensure that the substrate was uniformly covered with a thick gel. Preheating facilitated the solvent removal and increased the solution supersaturation, whereby nuclei formed on the substrate as seed crystals (Step 7 in Figure 1), after post-annealing at 800 °C and 1000 °C for 1 h in 5 × 10^−4^ Pa vacuum atmosphere. Finally, after annealing, the films were gradually cooled to room temperature (cooling rate ≈ 5 °C/min) within the furnace chamber to minimize thermal-stress-induced cracking, and a transparent film was obtained (Figure 1, Step 8).

The structural properties of the Ga_2_O_3_ thin films were measured by X-ray diffraction (XRD) in θ–2θ mode using Cu Kα radiation (Rigaku SmartLab 9 kW). Optical characterization was performed using an ultraviolet–visible–near-infrared spectrophotometer (PerkinElmer LAMBDA 1050) with a wavelength range of 200–2500 nm. The surface morphology of the samples was subsequently examined using field emission scanning electron microscopy (FE-SEM, SU8220 Hitachi, Tokyo, Japan).

## 3. Results and Discussion

### 3.1. Precursor Characterization

The transparent crystals (inset of Figure 2) produced during step (3) were meticulously blotted with clean paper and then dried at 60 °C. Then, the crystals were repeatedly ground into a powder for XRD analysis. Figure 2 shows the XRD pattern of the synthesized sample (blue line) alongside the standard powder diffraction file (PDF) data (International Centre of Diffraction Data/Powder Diffraction File [ICDD/PDF], No. 00-07-0018, red line). A comparison of the XRD patterns revealed a high degree of consistency between the synthesized powder and standard PDF data, which confirmed the successful synthesis of the target compound NH_4_Ga(SO_4_)_2_. The XRD pattern of the synthesized powder exhibited multiple distinct diffraction peaks in the 10° to 80° range. The positions and intensities of these diffraction peaks aligned closely with those in the standard PDF data, further verifying the purity of the sample and confirming that it possessed the correct crystal structure. Notably, the presence of several strong diffraction peaks in the 20° to 40° range indicated that the sample possessed good crystallinity. Furthermore, the absence of extraneous impurity peaks in the XRD pattern confirmed the high purity of the sample. A complete match between the diffraction peaks of the synthesized sample and the standard PDF data validated the effectiveness and reliability of this synthesis method. The inset in Figure 2 presents a photograph of the NH_4_Ga(SO_4_)_2_·12H_2_O crystals. The synthesized crystals exhibited a good transparency and regular morphology, which was consistent with the high crystallinity indicated by the XRD pattern. These results demonstrated that this synthesis method produced high-purity NH_4_Ga(SO_4_)_2_ powder and well-formed crystals.

From this, the precursor chemical reaction equation was derived:

(1) $$2Ga+3H_{2}SO_{4}\Rightarrow Ga_{2}{\left(SO_{4}\right)}_{3}+3H_{2}\;↑,$$

(2) $$Ga_{2}{\left(SO_{4}\right)}_{3}+{\left(NH_{4}\right)}_{2}SO_{4}+12H_{2}O\Rightarrow 2NH_{4}Ga{\left(SO_{4}\right)}_{2}\cdot 12H_{2}O,$$

(3) $$NH_{4}Ga{\left(SO_{4}\right)}_{2}\cdot 12H_{2}O\;\overset{160\;{}^{\circ}C}{\Rightarrow }\;NH_{4}Ga{\left(SO_{4}\right)}_{2}+12H_{2}O.$$

**Figure 3:**
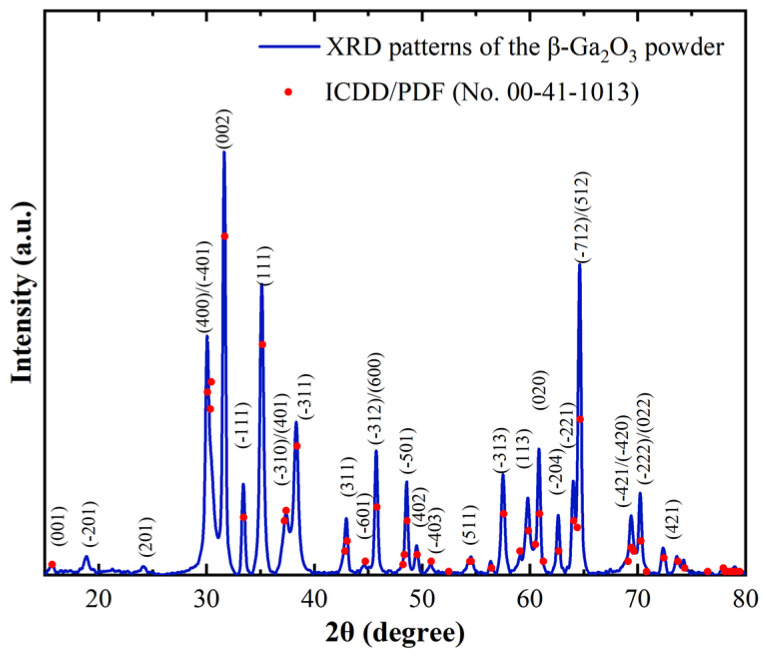
X-ray diffraction (XRD) patterns of the β-Ga_2_O_3_ powder prepared from NH_4_Ga(SO_4_)_2_·12H_2_O by sintering at 800 °C for 1 h. Note: ICDD/PDF stands for International Centre of Diffraction Data/Powder Diffraction File.

The NH_4_Ga(SO_4_)_2_ powder was initially heated at 160 °C for 30 min, followed by sintering in a quartz boat at 800 °C for 1 h. After grinding, the obtained powder was analyzed by XRD. Figure 3 presents the XRD patterns of the powder, which indicate that the diffraction peaks of the experimental powder aligned closely with the reference pattern from the ICDD/PDF (No. 00-41-1013) database. The results showed that the synthesized powder possessed the same crystal structure as the standard β-Ga_2_O_3_. The primary diffraction peaks were located at approximate 2θ values of 18.9°, 30.1°, 35.7°, 37.8°, 43.5°, 57.8°, and 63.4°, and these values corresponded well with the characteristic peaks of β-Ga_2_O_3_ reported in the literature [23,43]. Furthermore, the absence of other impurity peaks and the presence of several strong diffraction peaks confirmed the high purity and good crystallinity of the sample with no detectable secondary phases above the detection limit of XRD (~1–3 wt%). This indicates that the precursor fully decomposed to form the pure β-Ga_2_O_3_ phase. Therefore, the following chemical reaction could be inferred:

(4) $$2NH_{4}Ga{\left(SO_{4}\right)}_{2}\Rightarrow Ga_{2}O_{3}+4SO_{2}+2O_{2}+2NH_{3}+H_{2}O,$$

### 3.2. Analysis of the Surface Morphologies and Crystal Structure of the β-Ga_2_O_3_ Films on (0001) Sapphire

**Figure 4:**
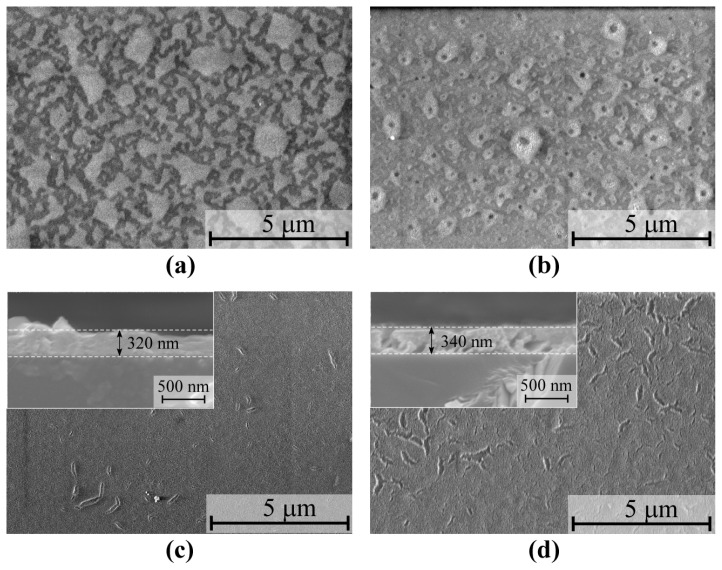
Surface scanning electron microscopy images of the β-Ga_2_O_3_ films on the (0001) sapphire substrate post-annealed at (**a**,**c**) 800 °C and (**b**,**d**) 1000 °C with repeated spin-coating and preheating cycles of 5 (**a**,**b**) and 10 (**c**,**d**). The insets of (**c**,**d**), respectively, present the cross-sectional SEM images of the films.

Figure 4a,b show the surface scanning electron microscopy (SEM) images of the β-Ga_2_O_3_ films subjected to spin-coating and preheating processes that were repeated for five cycles, followed by annealing at 800 °C and 1000 °C for 1 h. As shown in the SEM images, the surfaces of the β-Ga_2_O_3_ films annealed at 800 °C and 1000 °C, respectively, for 1 h exhibited a mottled and uneven texture, indicating that five spin-coating and preheating cycles were insufficient to form a continuous film. Therefore, the thickness of these films is difficult to determine. In contrast to the film annealed at 800 °C, the surface of the film annealed at 1000 °C exhibited small pores and spots, as illustrated in Figure 4b. This was possibly due to the occurrence of more intense diffusion and the aggregation of material particles at higher temperatures. This may also have been accompanied by some degree of volatilization or decomposition, leading to the transformation of the film surface from a mottled and uneven structure to a structure with small pores and depressions. After repeating the spin-coating and preheating process for ten cycles, the films were subjected to annealing at 800 °C and 1000 °C exhibited relatively continuous and flat surfaces, although some cracks were also observed on the surfaces of both films, as shown in Figure 4c,d. The insets of Figure 4c,d, respectively, present the cross-sectional SEM images of the β-Ga_2_O_3_ films. The film thicknesses were determined to be 320 nm and 340 nm. However, the surface of the film annealed at 800 °C was smoother, and it had fewer and finer cracks. In contrast, the surface of the film annealed at 1000 °C exhibited larger and denser cracks and was rougher. Recent theoretical developments in the growth mechanism analysis and the surface energy–kinetics coupling, particularly temperature-dependent aggregation-segregation phenomena at mesoscopic scales [44,45], provide valuable frameworks for understanding our experimental results. Therefore, the occurrence of cracks could be attributed to the volatilization of organic solvents and oxidative decomposition during high-temperature crystallization. The high annealing temperature enhanced the mobility of the atoms and laterally expanded the nucleus radius, causing the surface of the film to become slightly rougher. This may have been due to particle recombination induced by the greater surface free energy.

**Figure 5:**
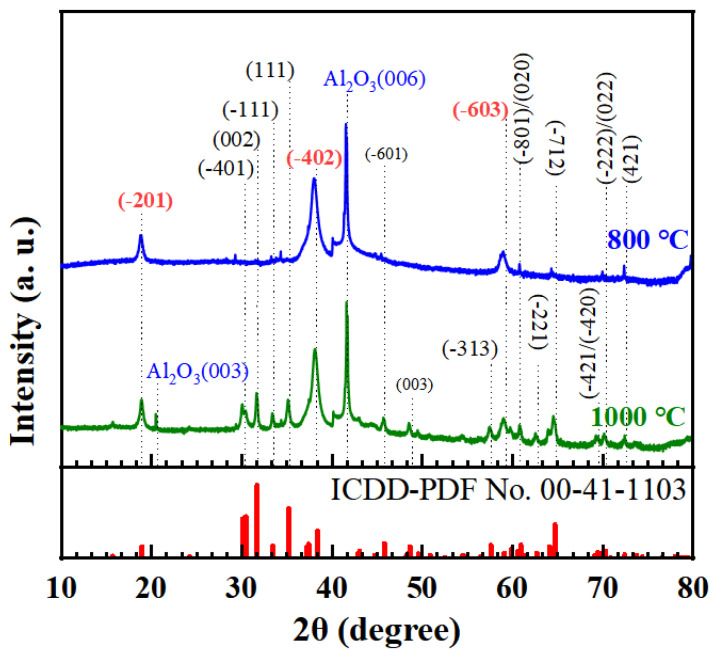
X-ray diffraction patterns of the β-Ga_2_O_3_ thin films prepared on the (0001) sapphire substrate at post-annealing temperatures of 800 °C and 1000 °C with ten spin-coating and preheating cycles. Note: ICDD/PDF stands for International Centre of Diffraction Data/Powder Diffraction File.

Figure 5 presents the XRD θ–2θ scan patterns of films on the (0001) sapphire substrate subjected to spin-coating and preheating processes repeated for ten cycles, followed by annealing at 800 °C and 1000 °C. The results exhibited excellent consistency with the patterns corresponding to β-Ga_2_O_3,_ as represented by ICDD-PDF No. 00-41-1103, confirming that the films were composed of β-Ga_2_O_3_. In addition to the diffraction peaks of the (0001) sapphire substrate, the XRD patterns clearly exhibited peaks at 18.9°, 38.4°, and 59.2°, which precisely corresponded to the (–201), (–402), and (–603) planes of β-Ga_2_O_3_, respectively. These characteristic peaks exhibited strong intensities, indicating that the films had a preferred orientation in the [–201] direction. For the films annealed at 1000 °C, in addition to the β-Ga_2_O_3_ (–201), (–402), and (–603) peaks parallel to the (0001) sapphire plane, diffraction signals from other planes, such as (–401), (–111), (111), and (–313), were also observed. This suggested the presence of grains from other orientations within the films, resulting in a complex polycrystalline structure. However, for the films annealed at 800 °C, with the exception of two small peaks corresponding to the (400) and (002) planes, peaks from other crystal planes were almost entirely absent. These results indicated that the films were predominantly oriented along the (–201) plane of β-Ga_2_O_3_, revealing the epitaxial relationship of [−201]β-Ga_2_O_3_//[0001]Al_2_O_3_. Thus, it was concluded that the β-Ga_2_O_3_ films in this work exhibited better crystalline qualities than other films.

### 3.3. Transmittance and Bandgap Study Using Ultraviolet–Visible–Near-Infrared Spectroscopy

**Figure 6:**
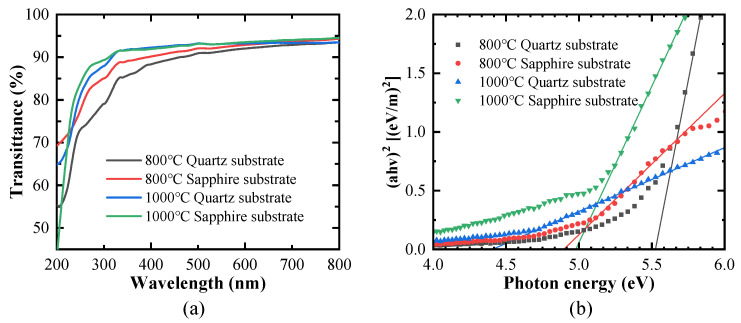
(**a**) The optical transmittance spectra of the β-Ga_2_O_3_ films on sapphire and quartz substrates post-annealed at 800 °C and 1000 °C for 1 h, and (**b**) the corresponding Tauc plot of the transmission curve for the films used to estimate the bandgap.

The transmittance spectra as a function of the wavelength for films prepared on different substrates and subjected to various post-annealing temperatures are shown in Figure 6a. These spectra clearly indicate that the transmittance of the films in the 400–800 nm wavelength range exceeded 80%. Additionally, all films exhibited significant absorption characteristics in the deep ultraviolet spectrum, with a sharp absorption edge observed near 250 nm. Under post-annealing temperatures of 800 °C and 1000 °C maintained for 1 h, the transmittance of the films on the quartz substrates was higher than that of the films on the sapphire substrates. This indicated that the quartz substrate materials were more favorable for enhancing transmittance. Regardless of the substrate used, the transmittance at the lower temperature (800 °C) was higher than at the higher temperature (1000 °C). This suggested that, as the post-annealing temperature increased, the transmittance also decreased. This decrease in transmittance may have been due to the deterioration of the optical properties of the material or the introduction of more optical losses at higher temperatures. Combined with the previous SEM analysis results, it is possible that the lower transmittance observed at higher annealing temperatures was due to the formation of larger and denser surface cracks in the films and their increased surface roughness. Furthermore, the XRD analysis indicated that the films subjected to high-temperature annealing exhibited a polycrystalline state, whereas the films annealed at 800 °C possessed a degree of preferred orientation; this suggests that films with a preferred orientation demonstrate a better transmittance performance.

To further analyze the optical properties, the optical bandgap was estimated from the transmittance spectra according to the following equation, known as the Tauc plot [46]:

(*αhν*)^1/*n*^ = *A*(*hν* − *E_g_*), (5)

where *h* is Plank’s constant, *ν* is the frequency, *α* is the absorption coefficient, and *A* is the proportional constant, where the power-law constant of *n* uses a value of 1/2 for direct transition and 2 for indirect transition. In this work, we assumed a direct transition, and the corresponding Tauc plot is shown in Figure 6b. Extrapolation of a straight line to the horizontal axis provided the *E_g_* for the films. As shown in Figure 6b, the bandgaps of the Ga_2_O_3_ film on the quartz substrate annealed at 800 °C and 1000 °C for 1 h were approximately 5.52 and 4.4 eV, respectively, when using ethylene glycol methyl ether as the solvent. In contrast, the Ga_2_O_3_ film on the sapphire substrate annealed at 800 °C for 1 h exhibited a bandgap of approximately 4.8 eV. When the film was subjected to annealing at 1000 °C for 1 h on the sapphire substrate, the bandgap was approximately 4.98 eV. This value was in good agreement with the experimental value of the β-Ga_2_O_3_ optical bandgap, indicating that sapphire was a more suitable substrate than quartz for the fabrication of Ga_2_O_3_ films.

## 4. Conclusions

In this study, β-Ga_2_O_3_ films were prepared on (0001) sapphire and quartz substrates through a simple and low-cost inorganic solution reaction sol–gel spin-coating method at a comparably low temperature (800 °C). NH_4_Ga(SO_4_)_2_·12H_2_O was refined using an inorganic solution reaction followed by separation. This approach allowed for the production of high-quality Ga_2_O_3_ thin films from low-grade Ga metals, thereby reducing the overall cost. The crystallinities and surface morphologies of the β-Ga_2_O_3_ films were significantly influenced by the number of spin-coating repetitions and the post-annealing temperature. Notably, the β-Ga_2_O_3_ film subjected to ten cycles of spin coating and annealed at 800 °C demonstrated a superior crystalline quality and smooth surface. Furthermore, these films exhibited a preferred (–201) orientation and revealed an epitaxial relationship characterized by [–201]β-Ga_2_O_3_//[0001]Al_2_O_3_. The β-Ga_2_O_3_ polycrystalline films were formed at an annealing temperature exceeding 800 °C. When the annealing temperature increased from 800 °C to 1000 °C, films with smooth surfaces and striped-like structures gradually transformed into rough crystallized films. All of the films showed a high transmittance of over 80% to ultraviolet and visible light with a wavelength above 400 nm. Specifically, the Ga_2_O_3_ films on the sapphire substrate and annealed at 800 °C and 1000 °C for 1 h exhibited optical energy bandgaps of approximately 4.8 and 4.98 eV, respectively. These results indicate that sapphire is a more suitable substrate than quartz for the fabrication of the Ga_2_O_3_ films, suggesting that this straightforward and efficient inorganic solution reaction method holds significant promise for the preparation of high-quality β-Ga_2_O_3_ films on high-temperature-resistant substrates.

## Data Availability

The datasets generated and/or analyzed during the current study are available from the corresponding authors upon reasonable request.
